# A conserved gene family encodes transmembrane proteins with fibronectin, immunoglobulin and leucine-rich repeat domains (FIGLER)

**DOI:** 10.1186/1741-7007-5-36

**Published:** 2007-09-13

**Authors:** Delicia L Munfus, Christopher L Haga, Peter D Burrows, Max D Cooper

**Affiliations:** 1 Division of Developmental and Clinical Immunology, University of Alabama at Birmingham, Birmingham, AL 35294-3300, USA; 2 Department of Microbiology, University of Alabama at Birmingham, Birmingham, AL 35294-3300, USA; 3 Department of Genetics, University of Alabama at Birmingham, Birmingham, AL 35294-3300,; 4 Department of Medicine, University of Alabama at Birmingham, Birmingham, AL 35294-3300, USA; 5 Department of Pediatrics University of Alabama at Birmingham, Birmingham, AL 35294-3300, USA

## Abstract

**Background:**

In mouse the cytokine interleukin-7 (IL-7) is required for generation of B lymphocytes, but human IL-7 does not appear to have this function. A bioinformatics approach was therefore used to identify IL-7 receptor related genes in the hope of identifying the elusive human cytokine.

**Results:**

Our database search identified a family of nine gene candidates, which we have provisionally named *fibronectin immunoglobulin leucine-rich repeat *(*FIGLER*). The *FIGLER 1–9 *genes are predicted to encode type I transmembrane glycoproteins with 6–12 leucine-rich repeats (LRR), a C2 type Ig domain, a fibronectin type III domain, a hydrophobic transmembrane domain, and a cytoplasmic domain containing one to four tyrosine residues. Members of this multichromosomal gene family possess 20–47% overall amino acid identity and are differentially expressed in cell lines and primary hematopoietic lineage cells. Genes for FIGLER homologs were identified in macaque, orangutan, chimpanzee, mouse, rat, dog, chicken, toad, and puffer fish databases. The non-human FIGLER homologs share 38–99% overall amino acid identity with their human counterpart.

**Conclusion:**

The extracellular domain structure and absence of recognizable cytoplasmic signaling motifs in members of the highly conserved FIGLER gene family suggest a trophic or cell adhesion function for these molecules.

## Background

Interleukin-7 (IL-7) is a non-redundant cytokine required for the generation of B and T lineage cells in mice [1-5]. Although IL-7 is essential for T cell development in humans, human B cell development is unaffected by the absence of IL-7 or its receptors [6-8]. Despite extensive research, the predicted IL-7 equivalent for human B lymphopoiesis has so far eluded identification. An important clue, provided by recent studies showing that human hematopoietic progenitors develop into mature B cells after transplantation in immunodeficient mice, suggests that the molecules essential for human B cell development are either present in the mouse or can be provided by the transplanted human cells [9,10]. In seeking a human B lymphopoietic cytokine/receptor pair, we reasoned that novel or orphan human receptors with structural features resembling those of the IL-7 receptor would be good candidates. A common feature of many cytokine receptors is the presence of Ig domains, fibronectin (FN) type III domains, and potential signaling capability [11]. Ig domains define members of the Ig superfamily, which is the largest family of mammalian cell surface molecules, comprising approximately half of the leukocyte cell surface glycoproteins [12]. FNIII domains are often found in molecules with adhesive function and can act as a spacer to ensure the correct positioning of functional sites [13].

Using bioinformatic searches for transmembrane proteins with Ig domains, FNIII domains, and signaling potential, nine human genes were identified that fulfilled the search criteria. These encode type I transmembrane glycoproteins, with 6–12 leucine-rich repeats (LRRs), one C2 Ig domain, one FNIII domain, a transmembrane domain, and a tyrosine containing cytoplasmic domain. The genes have been provisionally named *fibronectin immunoglobulin leucine-rich repeat *(*FIGLER*) *1–9*. In contrast to the known cytokine receptors, the predicted FIGLER molecules have a unique domain structure, marked by the N-terminal LRRs and an unusual genomic organization. Two previously described molecules that combine LRR, Ig and FNIII domains with unknown signaling capacities and function are included in this family, namely the photoreceptor-associated LRR superfamily member (PAL) and the neuronal leucine-rich repeat protein 3 (NLRR3) [14-22]. Here, we describe the features and expression patterns of the human FIGLER family members and identify multiple non-human orthologs.

## Results

### Identification of human FIGLER genes

**Figure 1:**
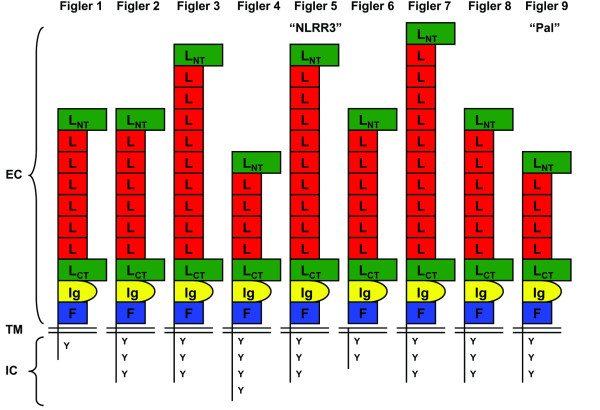
**Human FIGLER molecules**. Schematic representation of human FIGLER molecules 1–9 detailing extracellular motifs and intracellular tyrosines. L_NT_, N-terminal leucine-rich repeat; L, leucine-rich repeat; L_CT_, C-terminal leucine-rich repeat; Ig, C2 type Ig domain; FN, FN type III domain.

Over 3000 nucleotide and amino acid sequences of hypothetical proteins, as defined by the NCBI database, were analyzed by SMART and BLAST to determine domain structure and sequence similarity to known molecules. The initial screening of the human NCBI Genome Database led to the identification of a hypothetical gene that was predicted to encode a protein with IL-7 receptor-like structure in that it possessed both Ig and FNIII domains. The predicted amino acid sequence was then used to search NCBI's BLAST protein database, leading to the identification of eight other related molecules in humans (Figure 1 and Table 1). Based on analysis using the SMART database, each of these proteins is predicted to contain 6–12 LRR, one C2 Ig domain, one FNIII region, one hydrophobic transmembrane region and one to four cytoplasmic tyrosines. These molecules were provisionally named fibronectin immunoglobulin leucine-rich repeat (FIGLER) 1–9. Although the *FIGLER *genes are dispersed in the genome, the predicted amino acid sequences of the nine FIGLER molecules share 20–47% overall amino acid identity. Tyrosines are present in each of the FIGLER cytoplasmic regions, although they are not located within currently recognizable signaling motifs. Further analysis of the predicted amino acid sequences indicated that *FIGLER 5 *and *FIGLER 9 *correspond to the previously described *NLRR3 *and *Pal *genes [16,21,22].

**Table 1:** Percentage amino acid identity. Pairwise analysis of each FIGLER domain was performed using the Megalign CLUSTALW method algorithm, with FIGLER 1 serving as the index of comparison. Percent amino acid identities are indicated and aligned in relation to the FIGLER 1 domains. The identity percentage scoring employed here did not penalize for shortened cytoplasmic tails or the presence of < 8 LRRs.

Amino acid identity (%)								
FIGLER	2	3	4	5	6	7	8	9

LRR	53.1	33.6	28.1	32.1	62.2	35.0	56.6	27.9
Ig C2	55.4	36.9	31.8	38.5	64.6	41.5	52.3	32.9
FNIII	48.8	13.4	12.2	17.1	47.6	15.9	54.4	13.0
IC	39.2	6.8	13.2	13.5	29.7	17.6	36.5	11.4
EC	36.3	25.8	19.0	25.6	53.7	29.0	48.4	24.5
Overall	33.0	22.5	20.8	24.4	47.3	25.3	43.6	22.6

### Cellular expression of human FIGLER

**Figure 2:**
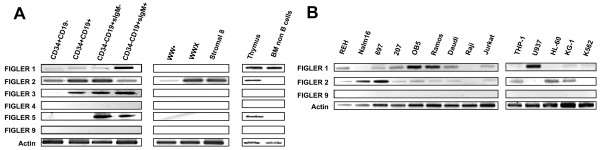
**Analysis of *FIGLER *expression in hematopoietic cells and cell lines**. (A) RT-PCR analysis of human *FIGLER *1–5 mRNA transcripts. Panel 1, primary bone marrow B lineage cells: CD34^+^CD19^-^, hemopoietic stem cells and other early hemopoietic cells; CD34^+^CD19^+^, pro-B cells; CD34^-^CD19^+^IgM^-^, pre-B cells; and CD34^-^CD19^+^IgM^+ ^B cells. Panel 2, bone marrow-derived stromal cell lines. Panel 3, primary thymus and bone marrow non-B lineage cells. (B) RT-PCR analysis of FIGLER 1, 2 and 9 mRNA transcripts in human pro-B (REH, Nalm16), pre-B (697, 207, OBS), B (Ramos, Daudi, Raji), T (Jurkat), myeloid (THP-1, U937, HL-60, KG-1), and erythoid (K562) cell lines. *FIGLER 9 *(Pal), a retina specific protein, served as a negative control and β-actin served as a loading control for all these analyses. PCR product identity was confirmed by sequencing as described in the Methods section.

As this study was directed initially toward identification of genes that might influence human B lymphopoiesis, RT-PCR analysis of *FIGLER *gene expression was performed on fetal bone marrow B lymphocyte subpopulations, bone marrow stromal cell lines, fetal thymus, and the non-B cell fraction in bone marrow. PCR products were cloned and sequenced to confirm their identity (data not shown). *FIGLER 9 *(*Pal*) served as a negative control gene in these experiments, as it was previously shown to be retina-specific (Figure 2A) [16]. The analysis indicated that *FIGLER 1*,*2*,*3*, and *5 *mRNA transcripts were expressed in primary B lineage cells. *FIGLER 1 *expression began at the immature sIgM^+ ^B cell stage. *FIGLER 2 *expression was detected at low levels in pro-B cells and in the CD34^+^CD19^- ^cells, a heterogeneous population that includes hematopoietic stem cells. *FIGLER 3 *expression was initiated at the pro-B cell stage, and *FIGLER 5 *expression began at the pre-B cell stage. *FIGLER 1 *and *5 *were also expressed by non-B lineage BM cells. Only *FIGLER 2 *was found to be expressed in human stromal cell lines. *FIGLER 4*,*6*,*7 *and *8 *could not be detected in any of the bone marrow-derived cells examined, despite multiple attempts and the use of three or more different gene-specific primer pairs per gene. To examine the *FIGLER *gene expression pattern further, we analyzed representative hemopoietic cell lines, B, T, myeloid and erythroid (Figure 2B). The up-regulation of *FIGLER 1 *expression observed as a function of normal B cell development was also observed in representative B lineage cell lines, although the OB5 pre-B cell line had relatively high levels compared to the normal pre-B cells. *FIGLER 1 *and *2 *were expressed weakly in the thymus and in the Jurkat T cell line, whereas they exhibited reciprocal expression patterns in myeloid cell lines. The K562 erythroid cell line did not express any of the *FIGLER *genes. Surprisingly, given their robust expression in primary cells of bone marrow origin, *FIGLER 3 *and *5 *were not detected in any of the cell lines tested, nor were *FIGLER 6*, *7*, and *8 *(data not shown). As the NCBI EST profile database did not contain bone marrow or spleen expression data for the *FIGLER *molecules, this constitutes the first evidence for expression of these molecules in the bone marrow and B lineage cells in particular.

### Identification of non-human FIGLER homologs

Putative *FIGLER *orthologs were found in macaque, chimpanzee, orangutan, mouse, rat, dog, chicken, toad, and puffer fish NCBI databases using the BLASTN algorithmic search for homology to the identified human *FIGLER *gene sequences (Tables 2, 3, 4, 5). Hidden Markov model searching of GenBank led to the identification of five potential new *FIGLER *orthologs in *Takifugu rubripes*, although little information is available about these molecules except amino acid sequence (data not shown).

**Table 2:** Percentage amino acid identity comparison of primate with human FIGLER

Amino acid identity with human FIGLER (%)					
Species	Macaque	*Pongo *(orangutan)	*Pan *(chimpanzee)		

FIGLER	2	5	1	8	9

LRR (Number)	100 (8)	99.1 (11)	100 (8)	62.7 (7)	97.7 (5)
Ig C2	98.8	100	100	68.2	100
FNIII	97.6	97.6	98.8	33.7	100
IC (Number of Tyr)	98.7 (2)	100 (2)	98.6 (1)	39.9 (5)	95.7 (2)
EC	99.4	99.7	99.8	49.7	98.7
Entire molecule	99.4	98.6	99.7	47.2	98.4

**Table 3:** Percentage amino acid identity comparison of rodent with human FIGLER

Amino acid identity with human FIGLER (%)										
Species	Mouse							Rat		

FIGLER	1	2	4	5	6	7	9	2	5	8

LRR (Number)	100 (8)	97.5 (8)	93.5 (6)	80.8 (10)	95.5 (18)	93.4 (12)	81.5 (6)	98.7 (8)	92.6 (10)	66.7 (8)
Ig C2	96.9	96.3	88.2	84.1	95.4	92.6	87.1	96.9	88.2	61.5
FNIII	95.2	98.8	95.1	67.1	98.8	82.9	83.5	93.1	92.8	62.5
IC (Number of Tyr)	85.1 (1)	84.4 (3)	58.3 (3)	76.0 (3)	94.9 (1)	84.0 (3)	67.1 (2)	82.2 (3)	64.4 (3)	41.4 (4)
EC	97.2	97.4	89.6	59.8	96.9	91.4	75.1	95.1	90.0	57.6
Entire molecule	95.8	94.9	56.3	54.0	96.5	87.7	71.6	92.7	87.2	56.5

**Table 4:** Percentage amino acid identity comparison of Tetra and Xenopus with human FIGLER

Amino acid identity with human FIGLER (%)						
Species	*Tetra *(puffer fish)				*Xenopus *(African clawed toad)	

FIGLER	2	3	8	9	2	3

LRR (Number)	78.7 (8)	74.8 (11)	67.8 (8)	54.3 (6)	68.3 (8)	64.7 (11)
Ig C2	75.8	69.6	63.6	56.5	70.3	69.6
FNIII	74.4	87.8	68.4	70.1	45.0	46.3
IC (Number of Tyr)	42.7 (5)	75.3 (5)	55.0 (2)	41.9 (3)	34.8 (4)	38.5 (7)
EC	77.4	83.1	62.0	49.7	39.0	60.1
Entire molecule	67.1	75.1	63.1	49.6	38.9	59.2

**Table 5:** Percentage amino acid identity comparison of dog, cow, and chicken with human FIGLER

Amino acid identity with human FIGLER (%)							
Species	Dog			Cow	Chicken		

FIGLER	2	5	8	1	4	8	9

LRR (Number)	99.1 (6)	97.9 (5)	60.1 (4)	99.2 (8)	64.4 (2)	97.8 (4)	68.9 (4)
Ig C2	98.4	90.0	59.7	100	84.3	95.5	77.1
FNIII	98.5	94.0	34.4	97.6	64.1	95.0	69.2
IC (Number of Tyr)	88.5 (2)	83.3 (3)	54.2 (6)	78.2 (1)	60.0 (3)	55.6 (1)	32.2 (1)
EC	98.9	95.2	65.5	97.2	52.5	96.0	54.4
Entire molecule	96.1	94.1	62.4	95.1	44.8	94.5	67.9

**Figure 3:**
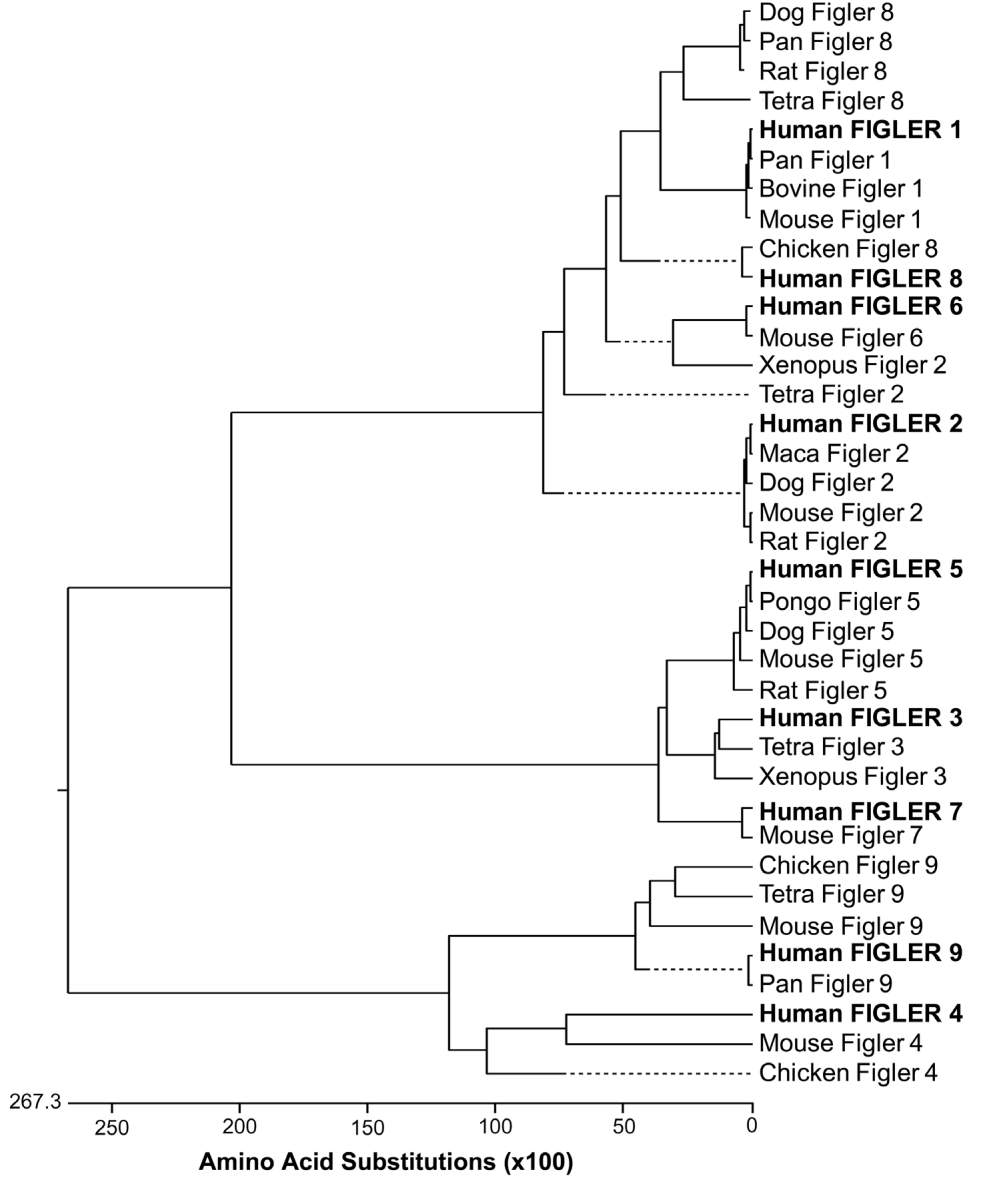
**Phylogenetic analysis of the *FIGLER *family of molecules**. Phylogenetic analysis of the molecule coding amino acid sequence region of the FIGLER family members. The CLUSTALW algorithm was used for multiple sequence alignment of divergent sequences after the variable length LRR regions were masked out.

As was the case for their human counterparts, these genes were found to be located on different chromosomes. All FIGLER orthologs contained 6–12 LRR, a single C2 type Ig domain, a FN type III domain, a hydrophobic transmembrane domain, and from one to seven cytoplasmic tyrosines. The predicted amino acid sequences of the FIGLER homologs shared 38–99% overall identity with their human counterpart. Phylogenetic tree analysis was performed to cluster the non-human FIGLER molecules to their nearest human FIGLER homolog (Figure 3). The alternative names, chromosomal locations, predicted amino acid length, and accession numbers of the human *FIGLER *and non-human *FIGLER *molecules are listed in Tables 6 and 7.

**Table 6:** Human and non-human FIGLER alternative names, chromosomal locations, predicted protein lengths and NCBI GenBank accession numbers

Species	Gene name (FIGLER)	Alternative name	Chromosome location	Predicted protein length (amino acids)	Protein accession number
*Homo Sapiens*	1	LRFN3	19q13.12	628	NP_078785
	2	LRFN2	6p21.2	789	NP_065788
	3	LRRN1	3p26.2	716	NP_065924
	4	FLJ44691	4q25	496	NP_940908
	5	LRRN3	7q31.1	708	NP_060804
	6	LRFN4	11q13.2	635	NP_076941
	7	LRRN5	1q32.1	713	NP_963924
	8	LRFN5	14q21.1	719	NP_689660
	9	PAL/LRRC21	10q23	623	NP_056428
*Macaca fascicularis*	2	LRFN2	-	789	BAB39323
*Mus musculus*	1	LRFN3	7	626	NP_780687
	2	mKIAA 1246	17	824	BAC65758
	4	-	3	679	XP_143529
	5	LRRN3/LRFN5	12	707	NP_034863
	6	LRFN4	19	636	NP_700437
	7	LRRN2	1	730	AAQ74241
	9	-	14	618	NP_666357
*Rattus norvegicus*	2	-	9q11	804	XP_236914
	5	NLRR3	6q21	707	NP_110483
	8	-	1q21	766	XP_344875

**Table 7:** Non-human FIGLER alternative names, chromosomal locations, predicted protein lengths and NCBI GenBank accession numbers

Species	Gene name (FIGLER)	Alternative name	Chromosome location	Predicted protein length (amino acids)	Protein accession number
*Gallus Gallus*	4	-	4	1009	XP_420649
	8	-	5	559	XP_421485
	9	-	6	884	XP_426489
*Tetraodon nigroviridis*	2	-	10	794	CAF99016
	3	-	11	715	CAG06728
	8	-	16	574	CAG08917
	9	-	17	660	CAF98662
*Pongo pygmaeus*	5	LRRN3/NLRR-3	-	708	CAH93434
*Pan troglodytes*	1	-	19	628	XP_524229
	8	-	19	795	XP_512991
	9	-	10	775	XP_521533
*Canis familiaris*	2	-	12	779	XP_538906
	5	-	14	708	XP_539523
	8	-	1	730	XP_541626
*Bos taurus*	1	LRFN3	18	628	NM_001076959
*Xenopus laevis*	2	LOC496079	-	722	AAH87496
	3	XNLRR-1	-	718	BAA28530

**Figure 4:**
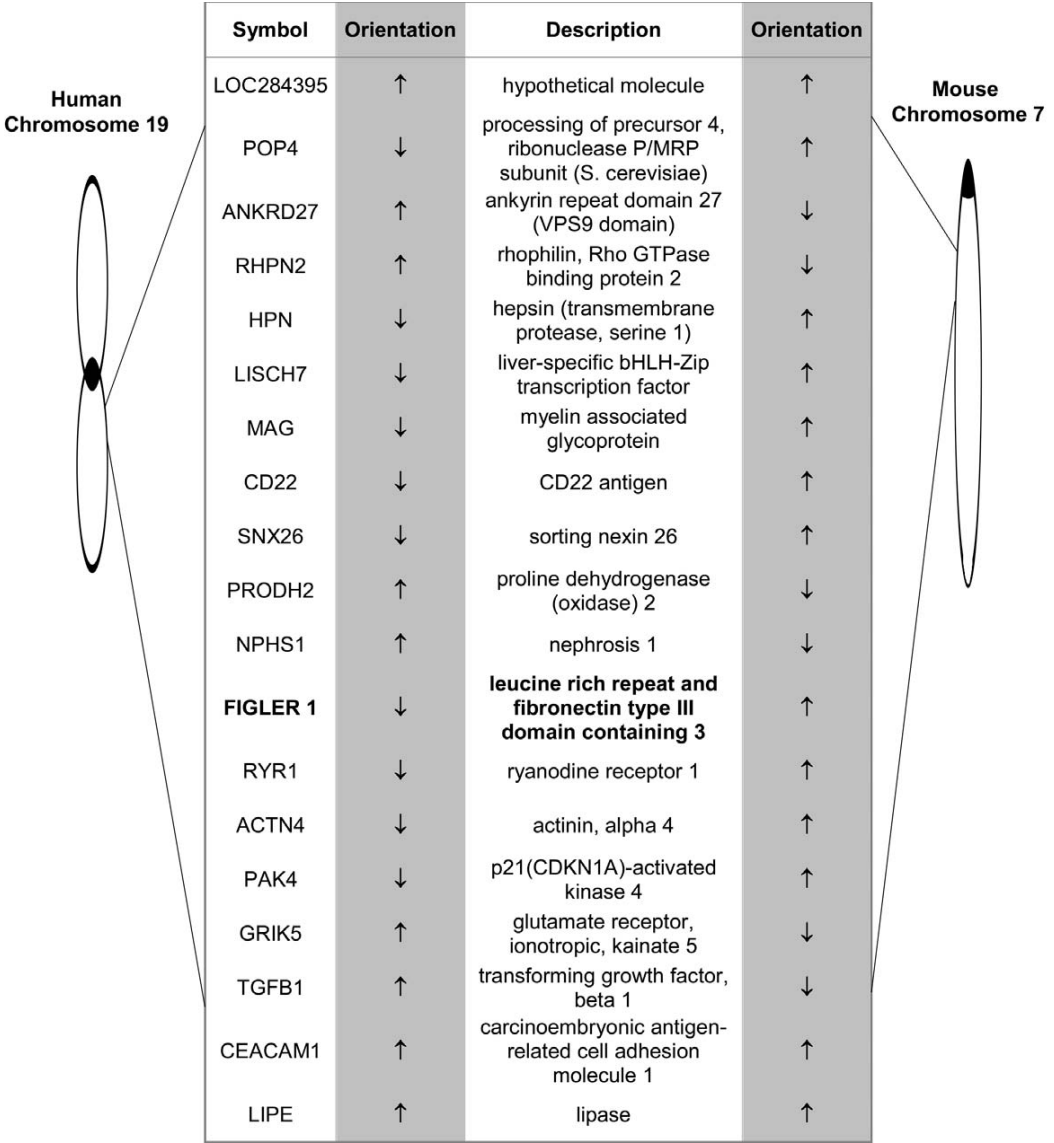
**Human/mouse homology map for *FIGLER 1***. The chromosomal locations and transcriptional orientation of the human and mouse *FIGLER 1 *and flanking genes were determined using the NCBI GenBank Mapview Database. Black ovals denote the centromere.

The chromosomal regions of the human and mouse *FIGLER *genes were compared to further examine their relatedness. The positions of neighboring upstream and downstream genes were found to be highly conserved, but the regions containing the mouse *FIGLER *genes were inverted relative to the orientation of human *FIGLER *chromosomal regions. This is illustrated for *FIGLER 1 *and mouse *FIGLER 1 *(Figure 4). By contrast, the non-mouse *FIGLER *genes have the same chromosomal orientation as their human *FIGLER *counterparts (data not shown).

**Figure 5:**
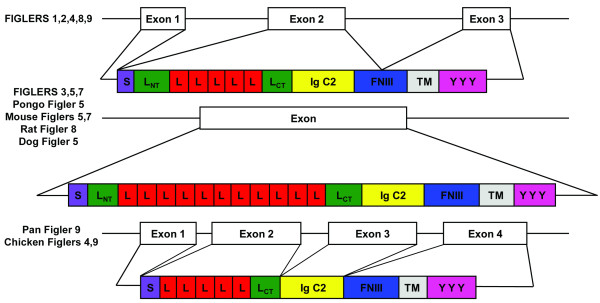
**Predicted genomic structure and protein product of human and non-human FIGLER molecules**. S, Signal Peptide; L_NT_, N-terminal leucine-rich repeat; L, leucine-rich repeat domain; L_CT_, C-terminal leucine-rich repeat, Ig, C2 type Ig domain; FNIII, FN type III domain; TM, hydrophobic transmembrane domain; Y, tyrosine in predicted cytoplasmic domain. Non-human *FIGLER *genes not listed in this schematic have the same three exon structure as human FIGLER 1, 2, 4, 8, and 9.

As the known *NLRR3 *(*FIGLER 5*) and *Pal *(*FIGLER 9*) genes have been shown to have an unusual three exon structure, we analyzed the predicted exon structure of the entire *FIGLER *gene family (Figure 5). Most of the human *FIGLER *and non-human *FIGLER *genes have the same atypical 3 exon structure, in which exon 1 contains the 5' untranslated region; exon 2 encodes the signal peptide, LRR, Ig domain, and part of the FNIII domain; and exon 3 encodes the remaining FNIII region, transmembrane region, cytoplasmic tail, and 3' untranslated region. Human *FIGLER 3*, *5*, and 7, chimpanzee *FIGLER 5*, mouse *FIGLER 5 *and *7*, rat *FIGLER 8*, and dog *FIGLER 5 *are encoded by a single exon. Interestingly, chimpanzee and chicken *FIGLER 9 *and chicken FIGLER 4 have a more standard exon organization, in which exon 1 contains the 5'untranslated region, exon 2 encodes the LRRs, exon 3 contains the Ig domain, and exon 4 encodes the FNIII region, transmembrane domain, cytoplasmic tail and 3'UTR.

## Discussion

The nine human orphan receptors identified here were named FIGLER to emphasize their unique combination of FN, Ig, and LRR domains. The tyrosines present in their cytoplasmic regions may indicate signaling potential, although they were not found to be embedded in known consensus signaling motifs. The *FIGLER *genes encode molecules that share 20–47% amino acid identity and similar structure despite their widespread distribution in the genome. Although our initial goal was to identify a novel IL-7-like receptor, the FIGLER molecules differ from known cytokine receptors in that they have LRR domains on the N-terminal end of the protein. The LRRs are characterized by specific leucine and asparagine residue spacing and are found in proteins with diverse functions and cellular distribution [14,15,17,21,22]. LRRs also are found in other molecules that are expressed by B cells, such as Toll-like receptors 9 and 10 [23]. As we sought to identify molecules that could potentially influence human B lymphopoiesis, *FIGLER *expression was analyzed in hematopoietic and lymphoid tissues. *FIGLER 1*,*3*, and *5 *mRNA expression was found to be up-regulated as a function of human B cell development, whereas *FIGLER 2 *was expressed throughout development and in non-B lineage cells.

The identification of *FIGLER *relatives in macaque, cow, chimpanzee, orangutan, mouse, rat, dog, chicken, toad and puffer fish that share 38–99% amino acid identity to their human counterparts indicates that this is a conserved gene family. All of the members encode type I transmembrane proteins, and each gene is located on a different chromosome, except for the chimpanzee *FIGLER 1 *and *8*, which are both located on chromosome 19. Based on human *FIGLER *and mouse *FIGLER *gene chromosomal analysis, it is likely that the human and non-human *FIGLER *genes are homologs that were derived from common ancestral genes. Intriguingly, the mouse FIGLER genes are located on inverted chromosomal segments relative to their human syntenic regions, although *FIGLER *genes in other species have the same orientation as their human counterparts.

As the known *NLRR3 *(*FIGLER 5*) and Pal (*FIGLER 9*) genes have been shown to have an unusual predicted exon structure, we determined the exon structure of the entire *FIGLER *gene family [16,21,22]. Most of the human *FIGLER *and non-human *FIGLER *genes have the same atypical 3 exon structure, in which exon 1 contains the 5' untranslated region; exon 2 encodes the signal peptide, LRR, Ig domain, and part of the FNIII domain; and exon 3 encodes the remaining FNIII region, transmembrane region, cytoplasmic tail, and 3' untranslated region. Ig domains are almost universally encoded by a single exon, which in part is thought to have accounted for their ready dispersal in the genome and widespread utilization in cell surface glycoproteins with multiple functions. Human *FIGLER 3*, *5*, and *7*, chimpanzee *FIGLER 5*, mouse *FIGLER 5*, mouse *FIGLER 7*, rat *FIGLER *8, and dog *FIGLER 5 *are all intronless genes, suggesting their integration into the genome via reverse transcription. Nevertheless we have demonstrated cellular expression for all but *FIGLER 4 *and *6–8*, and all of these genes are predicted to encode an intact open reading frame. Finally, the chimpanzee and chicken *FIGLER 9 *genes and chicken *FIGLER 4 *have a more typical organization, and the Ig domain is encoded by a separate exon.

## Conclusion

The functions of the *FIGLER *gene products are presently unknown. FIGLER 5 and 9 are identical to the previously identified Pal and NLRR3. Pal is a type 1 transmembrane protein that is preferentially expressed in the retina, where it is up-regulated during photoreceptor outer segment development. As for the other FIGLER family members, Pal lacks recognizable signaling motifs in its cytoplasmic tail and is predicted to act as a trophic factor receptor or adhesion molecule [16]. NLRRs were identified through screening of a mouse brain cDNA library, and homologs have been found in *Xenopus*, rat, and human brain tissue [18-20,24,25]. All of the NLRR members are expressed predominately in the central nervous system [24]. Of the three known mouse NLRRs, only NLRR3 is up-regulated with brain injury, thereby suggesting its involvement in injury recognition or repair [26]. While several of the FIGLER molecules described here are expressed in B lineage cells and the expression of FIGLER 1, 3, and 5 is developmentally regulated within this lineage, the possibility that these molecules are involved in the interaction between B lineage cells with the supportive bone marrow microenvironment remains speculative.

## Methods

### Database and search strategies

The nucleotide sequence, amino acid sequence and domain structure of the human IL-7 receptor α chain (NM_002185) was used in the basic local alignment search tool (BLAST) and simple modular architecture research tool (SMART) database searches for novel IL-7 receptor α chain relatives [27-29]. The National Center for Biotechnology Information (NCBI), European Molecular Biology Laboratory (EMBL), Ensembl, and DNA database in Japan were all queried, including the expressed sequence tags (EST) and high throughput genomic sequences (HTGS) databases. The predicted human *FIGLER *sequences were used to identify homologs in other species using BLASTN algorithmic and hidden Markov model (HMM) searches of the NCBI and GenBank databases. HMM hits were filtered through Pfam according to their domain composition. The NCBI genome database was used to align the predicted mRNA sequences to the genomic sequences and determine intron-exon boundaries. The human BLAST-like alignment tool (BLAT) database (University of California at Santa Cruz) and Megalign CLUSTALW method algorithm (Windows version 3.12e; DNASTAR, Madison, WI, USA) were used to design primers and determine amino acid identity among the newly identified FIGLER molecules. The molecular coding amino acid sequence region phylogenetic tree analysis of the FIGLER molecules as listed in the GenBank database was conducted using the CLUSTALW method algorithm of Megalign [30]. The variable length LRR regions were masked out during phylogenetic analysis in order to compensate for sequence length.

### Cell lines

Human stromal cell lines included the WW, WWX, and stromal 8 lines, which were derived from bone marrow samples in our laboratory. B lineage cell lines were REH and Nalm 16 (pro B cell); 697, 207, and OB5 (pre B cell); and Ramos, Daudi, and Raji (B cell). Myeloid cell lines included THP-1 (monocytic), HL-60 (promyelocytic), U937, and KG-1 (myelocytic). The Jurkat T cell line and K562 erythroid cell lines were also used in this analysis.

### Human bone marrow and thymus primary cell isolation

Human adult bone marrow was obtained from resected ribs of healthy renal transplant donors in accordance with policies established by the University of Alabama at Birmingham Institutional Review Board. The resected ribs were processed within 24 h of obtainment, and lymphoid cells were isolated by Ficoll-Hypaque gradient centrifugation (Mediatech, Herndon, VA, USA). B lineage cells were isolated from the bone marrow samples using a MACS B cell Isolation Kit (Miltenyl Biotec, Auburn, CA, USA). The isolated primary B lineage cells were resuspended in FACS buffer (FACS buffer: PBS plus 2% FBS) and incubated for 20 min at 4°C with CD34-APC, CD19-PE, and IgM-FITC labeled antibodies (BD Biosciences, Palo Alto, CA, USA). The cells were then sorted based on their differential CD34, CD19, and IgM expression into four B lineage populations: CD34^+^19^-^, CD34^+^19^+^, CD34^-^19^+^IgM^-^, and CD34^-^19^+^IgM^+^, using a MoFlo instrument (Cytomation, Fort Collins, CO, USA). Human fetal bone marrow and thymus samples used for RT-PCR analysis were obtained in accordance with policies established by the University of Alabama at Birmingham Institutional Review Board. Lymphocytes from 12-to 29-week fetal bone marrow and thymus were obtained by sedimentation at 100 *g *over a lymphocyte separation medium for 30 min at room temperature. The cells recovered at the interface were washed in PBS containing 5% FCS and 2 to 4 × 10^7 ^cells. B lineage cells were isolated from the bone marrow and thymus samples using a MACS B cell Isolation Kit.

### Reverse transcription (RT)-PCR

Total RNA was isolated from isolated populations of primary fetal bone marrow B lineage cells, primary fetal thymic T lineage cells, hematopoietic cell lines, and stromal cell lines using TRIzol (Invitrogen, Carlsbad, CA, USA). cDNA was generated from the isolated mRNA using Superscript First Strand Synthesis System for RT-PCR, per the manufacturer's suggestions (Invitrogen). The following gene specific primers were used to amplify each gene via PCR using Platinum Taq DNA Polymerase (Invitrogen): FIGLER 1, forward 5'-CTGCTAGGCAACTCAAGC-3' and reverse 5'-GATAGGCCGCTGATCCG-3'; FIGLER 2, forward 5'-GCTACTTCTGGCATGTGC-3' and reverse 5'-ACCACTGTCCTGAGATGT-3'; FIGLER 3, forward 5'-CAGTACAGCCCTTGCTG-3' and reverse 5'-CCACATGTAATAGCTTG-3'; FIGLER 4, forward 5'-CTCGTGGTGACCAGTACT-3' and reverse 5'-AGCTTCTGTCACGTCTGC-3'; FIGLER 5, forward 5'-CAGCAATGCTCTCAGTGC-3' and reverse 5'-TCGAGCACTTTGCGCAG-3'; FIGLER 9, forward 5'-TCTCAATGCAGCTGCAGC-3' and reverse 5'-GCTGGCACATCTCAGTTC-3'. Each amplification reaction underwent an initial denaturation of 94°C for 2.5 min, followed by 35 cycles of annealing at 55°C for 1 min, extension at 72°C for 1 min, denaturation at 94°C for 1 min, and a final extension at 72°C for 10 min. The PCR products were separated by agarose gel electrophoresis, and their identity was verified via sequencing after cloning into a TOPO TA pCR2.1 Vector (Invitrogen). DNA sequencing was accomplished for both strands by the dideoxy chain termination method using Thermo Sequenase (Amersham Biosciences Corp, Piscataway, NJ, USA), per the manufacturer's suggestions, and an automated sequencer (LiCor, Lincoln, NE, USA).

## Competing interests

The author(s) declares that there are no competing interests.

## Authors' contributions

DLM conducted the primary database searches, analysis, RT-PCR experiments, and manuscript preparation. CLH contributed to database searches, analysis, experimental design, interpretation, and manuscript preparation. PDB contributed to the experimental design, analysis, interpretation, and manuscript preparation. MDC contributed to the experimental design, interpretation, and manuscript preparation. All authors have read and approved the final manuscript.

## References

[B1] Carvalho TL, Mota-Santos T, Cumano A, Demengeot J, Vieira P (2001). Arrested B lymphopoiesis and persistence of activated B cells in adult interleukin 7-/- Mice. J Exp Med.

[B2] Grabstein KH, Waldschmidt TJ, Finkelman FD, Hess BW, Alpert AR, Boiani NE, Namen AE, Morrissey PJ (1993). Inhibition of murine B and T lymphopoiesis *in vivo *by an anti-interleukin 7 monoclonal antibody. J Exp Med.

[B3] Miller JP, Izon D, DeMuth W, Gerstein R, Bhandoola A, Allman D (2002). The earliest step in B lineage differentiation from common lymphoid progenitors is critically dependent upon interleukin 7. J Exp Med.

[B4] Valenzona HO, Dhanoa S, Finkelman FD, Osmond DG (1998). Exogenous interleukin 7 as a proliferative stimulant of early precursor B cells in mouse bone marrow: efficacy of IL-7 injection, IL-7 infusion and IL-7-anti-IL-7 antibody complexes. Cytokine.

[B5] von Freeden-Jeffry U, Vieira P, Lucian LA, McNeil T, Burdach SE, Murray R (1995). Lymphopenia in interleukin (IL)-7 gene-deleted mice identifies IL-7 as a nonredundant cytokine. J Exp Med.

[B6] Prieyl JA, LeBien TW (1996). Interleukin 7 independent development of human B cells. Proc Natl Acad Sci USA.

[B7] Puel A, Leonard WJ (2000). Mutations in the gene for the IL-7 receptor result in T(-)B(+)NK(+) severe combined immunodeficiency disease. Curr Opin Immunol.

[B8] Puel A, Ziegler SF, Buckley RH, Leonard WJ (1998). Defective IL7R expression in T(-)B(+)NK(+) severe combined immunodeficiency. Nat Genet.

[B9] Hiramatsu H, Nishikomori R, Heike T, Ito M, Kobayashi K, Katamura K, Nakahata T (2003). Complete reconstitution of human lymphocytes from cord blood CD34+ cells using the NOD/SCID/gammacnull mice model. Blood.

[B10] Li C, Ando K, Kametani Y, Oki M, Hagihara M, Shimamura K, Habu S, Kato S, Hotta T (2002). Reconstitution of functional human B lymphocytes in NOD/SCID mice engrafted with *ex vivo *expanded CD34(+) cord blood cells. Exp Hematol.

[B11] Barclay AN, Brown MH, Alex Law SK, McKnight AJ, Tomlinson MG, van der Merwe PA (1997). The Leucocyte Antigen: Facts Book.

[B12] Holness CL, Simmons DL (1994). Structural motifs for recognition and adhesion in members of the immunoglobulin superfamily. J Cell Sci.

[B13] Potts JR, Campbell ID (1994). Fibronectin structure and assembly. Curr Opin Cell Biol.

[B14] Bormann P, Roth LW, Andel D, Ackermann M, Reinhard E (1999). zfNLRR, a novel leucine-rich repeat protein is preferentially expressed during regeneration in zebrafish. Mol Cel Neurosc.

[B15] Fukamachi K, Matsuoka Y, Kitanaka C, Kuchino Y, Tsuda H (2001). Rat neuronal leucine-rich repeat protein-3: cloning and regulation of the gene expression. Biochem Biophys Res Commun.

[B16] Gomi F, Imaizumi K, Yoneda T, Taniguchi M, Mori Y, Miyoshi K, Hitomi J, Fujikado T, Tano Y, Tohyama M (2000). Molecular cloning of a novel membrane glycoprotein, pal, specifically expressed in photoreceptor cells of the retina and containing leucine-rich repeat. J Neurosci.

[B17] Hayata T, Uochi T, Asashima M (1998). Molecular cloning of XNLRR-1, a *Xenopus *homolog of mouse neuronal leucine-rich repeat protein expressed in the developing *Xenopus *nervous system. Gene.

[B18] Kajava AV (2001). Review: proteins with repeated sequence – structural prediction and modeling. J Struct Biol.

[B19] Kobe B, Deisenhofer J (1994). The leucine-rich repeat: a versatile binding motif. Trends Biochem Sci.

[B20] Malim MH, Bohnlein S, Hauber J, Cullen BR (1989). Functional dissection of the HIV-1 Rev trans-activator–derivation of a trans-dominant repressor of Rev function. Cell.

[B21] Taguchi A, Wanaka A, Mori T, Matsumoto K, Imai Y, Tagaki T, Tohyama M (1996). Molecular cloning of novel leucine-rich repeat proteins and their expression in the developing mouse nervous system. Brain Res.

[B22] Taniguchi H, Tohyama M, Takagi T (1996). Cloning and expression of a novel gene for a protein with leucine-rich repeats in the developing mouse nervous system. Brain Res.

[B23] Bourke E, Bosisio D, Golay J, Polentarutti N, Mantovani A (2003). The toll-like receptor repertoire of human B lymphocytes: inducible and selective expression of TLR9 and TLR10 in normal and transformed cells. Blood.

[B24] Andrade MA, Perez-Iratxeta C, Ponting CP (2001). Protein repeats: structures, functions, and evolution. J Struct Biol.

[B25] Takahashi N, Takahashi Y, Putnam FW (1985). Periodicity of leucine and tandem repetition of a 24-amino acid segment in the primary structure of leucine-rich alpha 2-glycoprotein of human serum. Proc Natl Acad Sci USA.

[B26] Ishii N, Wanaka A, Tohyama M (1996). Increased expression of NLRR-3 mRNA after cortical brain injury in mouse. Brain Res.

[B27] Altschul SF, Gish W, Miller W, Myers EW, Lipman DJ (1990). Basic local alignment search tool. J Mol Biol.

[B28] Letunic I, Goodstadt L, Dickens NJ, Doerks T, Schultz J, Mott R, Ciccarelli F, Copley RR, Ponting CP, Bork P (2002). Recent improvements to the SMART domain-based sequence annotation resource. Nucleic acids Res.

[B29] Schultz J, Milpetz F, Bork P, Ponting CP (1998). SMART, a simple modular architecture research tool: identification of signaling domains. Proc Natl Acad Sci USA.

[B30] Thompson JD, Higgins DG, Gibson TJ (1994). CLUSTAL W: improving the sensitivity of progressive multiple sequence alignment through sequence weighting, position-specific gap penalties and weight matrix choice. Nucleic Acids Res.

